# Adolescent Gender Differences in Cognitive Control Performance and Functional Connectivity Between Default Mode and Fronto-Parietal Networks Within a Self-Referential Context

**DOI:** 10.3389/fnbeh.2018.00073

**Published:** 2018-04-23

**Authors:** Gabriela Alarcón, Jennifer H. Pfeifer, Damien A. Fair, Bonnie J. Nagel

**Affiliations:** ^1^Department of Psychiatry, University of Pittsburgh, Pittsburgh, PA, United States; ^2^Department of Psychology, University of Oregon, Eugene, OR, United States; ^3^Department of Behavioral Neuroscience, Oregon Health & Science University, Portland, OR, United States; ^4^Department of Psychiatry, Oregon Health & Science University, Portland, OR, United States; ^5^Advanced Imaging Research Center, Oregon Health & Science University, Portland, OR, United States

**Keywords:** adolescence, self-referential processing, cognitive control, gender differences, co-rumination, functional magnetic resonance imaging, default mode network, fronto-parietal network

## Abstract

Ineffective reduction of functional connectivity between the default mode network (DMN) and frontoparietal network (FPN) during cognitive control can interfere with performance in healthy individuals—a phenomenon present in psychiatric disorders, such as depression. Here, this mechanism is studied in healthy adolescents by examining gender differences in task-regressed functional connectivity using functional magnetic resonance imaging (MRI) and a novel task designed to place the DMN—supporting self-referential processing (SRP)—and FPN—supporting cognitive control—into conflict. Compared to boys, girls showed stronger functional connectivity between DMN and FPN during cognitive control in an SRP context (*n* = 40; boys = 20), a context that also elicited more errors of omission in girls. The gender difference in errors of omission was mediated by higher self-reported co-rumination—the extensive and repetitive discussion of problems and focus on negative feelings with a same-gender peer—by girls, compared to boys. These findings indicate that placing internal and external attentional demands in conflict lead to persistent functional connectivity between FPN and DMN in girls, but not boys; however, deficits in performance during this context were explained by co-rumination, such that youth with higher co-rumination displayed the largest performance deficits. Previous research shows that co-rumination predicts depressive symptoms during adolescence; thus, gender differences in the mechanisms involved with transitioning from internal to external processing may be relevant for understanding heightened vulnerability for depression in adolescent girls.

## Introduction

The adolescent period is characterized by pronounced maturation of social, cognitive and biological processes that lead to distinct developmental outcomes for girls and boys. These outcomes include normative, as well as psychopathological gender differences, like higher rates of depression in adolescent girls compared to boys. Understanding normative developmental gender differences provides a context through which gender differences in psychopathology may be interpreted. A changing social landscape informs the development of adolescent self-referential processing (SRP), or the act of relating information to the self, to re-shape self-identity (Coleman and Hendry, 1990). Gender differences in the types of social experiences boys and girls will encounter (Rose and Rudolph, 2006) may lead to gender differences in SRP that differentially inform self-identity. Moreover, increasing autonomy and academic demands help shape maturation of adolescent cognitive control (Luna, 2009). Although gender differences in cognitive control performance appear to be minimal (Gur et al., 2012; Satterthwaite et al., 2015), there are significant gender differences in neural activation during cognitive control functioning (Mueller, 2011). Importantly, the interaction between neural networks that support SRP (i.e., default mode network, DMN) and cognitive control (i.e., frontoparietal network, FPN) are disrupted in certain psychiatric disorders, including major depressive disorder (MDD; Wagner et al., 2006, 2015; Bartova et al., 2015), which disproportionately affects girls and young women (Hedden et al., 2015). The intersection of SRP and cognitive control during healthy adolescence has not been studied but is relevant for comprehending normative developmental baselines and uncovering potential neural mechanisms that function as gender-specific vulnerability factors for MDD.

A developmental increase in valuation of peers and social feedback during adolescence informs self-identity formation (Nelson et al., 2005; Steinberg, 2005). Compared to boys, girls place a higher value on social goals, report stronger same-gender peer attachments and are rated as more prosocial (Rose and Rudolph, 2006). Studies of healthy development indicate that adolescent social behavior and self-identity are shaped by peer feedback (Harter et al., 1998; Harter, 1999; Berzonsky and Adams, 2003; Pfeifer et al., 2009). Indeed, neuroimaging research suggests that adolescents may reflexively incorporate perceived opinions of others into their direct evaluation of self, as indicated by activation of perspective-taking brain regions during SRP (Pfeifer et al., 2009). SRP is typically characterized by activation of medial prefrontal cortex (mPFC) and posterior cingulate cortex (PCC; Gusnard et al., 2001; Kelley et al., 2002), key nodes of the DMN (Raichle, 2015). Moreover, perceived social evaluations of close peers during SRP elicits robust activation of the ventral striatum, in addition to the mPFC (Jankowski et al., 2014). Although adolescent girls are more prosocially oriented than boys (Rose and Rudolph, 2006), they also display more social-evaluative concern (Rudolph and Conley, 2005), which may be reflected in their self-evaluations, as they reflexively incorporate perceived opinions of their peers. Therefore, not only it is important to assess prosocial orientation when studying gender differences in adolescent SRP, but also to understand potentially maladaptive patterns of engaging with peers and how adolescents perceive themselves (see “Participant Characterization” section).

In addition to the development of social cognition and self-identity formation, cognitive control processes undergo substantial maturation during adolescence, in part due to the protracted rates of development of parietal and prefrontal cortices (Tamnes et al., 2017) that likely reflect synaptic pruning and neuronal specialization (Selemon, 2013), as well as intracortical myelination (Seldon, 2007). The frontal and parietal cortices, which make up the FPN, are the most consistently reported cortical regions supporting cognitive control (Cole and Schneider, 2007; Dosenbach et al., 2008). Although previous work suggests a lack of gender difference in cognitive control performance (Gur et al., 2012; Satterthwaite et al., 2015), studies report gender differences in neural activation during affectively laden cognitive control tasks. For example, studies examining brain activation during cognitive control in negative affective contexts have found that adolescent and adult males display stronger activation in the FPN than females (Koch et al., 2007; Cservenka et al., 2015). Therefore, gender differences in cognitive control brain response may only emerge in affective and/or salient contexts.

The interaction of SRP and cognitive control has not been studied in healthy adolescents; however, the interaction of the networks that support SRP and cognitive control are well described. The DMN and FPN are neural networks that support distinct functions of the brain, characterized by inwardly- and outwardly-directed attention, respectively (Greicius et al., 2003; Fox et al., 2005; Fransson, 2005; De Luca et al., 2006). During effortful cognitive control, these networks become segregated, or anti-correlated, in order to shift cognitive resources from DMN- to FPN-supported functions (Kelly et al., 2008; Hampson et al., 2010; Keller et al., 2015). In healthy adults, the DMN fails to deactivate during cognitive control trials that are preceded by an SRP condition; in fact, activation of the DMN is more robust in individuals with higher depression scores (Wagner et al., 2013). Adolescence is a prime developmental period to investigate this mechanism given the dynamic maturation of both SRP and cognitive control during this time frame.

A growing number of studies suggest that baseline or spontaneous neural activity continues during tasks and that task-related activation represents a combination of spontaneous activity and responses to task stimuli (Arfanakis et al., 2000; Fox et al., 2006; Fair et al., 2007; Gavrilescu et al., 2008; Zhang and Li, 2010, 2012). Thus, by regressing task signal from a time course, the spontaneous activity remains. However, the resulting functional connectivity patterns of task-regressed and resting state time courses are not identical, which may reflect “contamination” of task-regressed signal by task processes (Arfanakis et al., 2000), or nonlinear effects of a task that are not removed with linear regression (Fair et al., 2007). Alternatively, it is possible that task engagement acutely and persistently alters functional connectivity (Lowe et al., 2000; Hampson et al., 2004; Fransson, 2006; Fair et al., 2007), which is what will be measured in the current study. Specifically, we will measure the alterations in the functional connectivity of the underlying spontaneous activity following regression of task-related activity. The current study examined this mechanism by measuring gender differences in functional connectivity of the networks that support SRP and cognitive control—DMN and FPN, respectively—during cognitive control trials in SRP (vs. Control) conditions. We hypothesized that based on literature suggesting that adolescent girls demonstrate a stronger prosocial orientation and social-evaluative concern than boys (Rudolph and Conley, 2005; Rose and Rudolph, 2006), and that adolescents reflexively incorporate perceived peer opinions into their self-identity (Pfeifer et al., 2009), girls would: (1) report higher prosocial orientation, more negative self-perceptions and more maladaptive patterns of peer engagement (see “Participant Characterization”). Furthermore, based on studies suggesting that gender differences in cognitive control brain response may only emerge in affective contexts (Koch et al., 2007; Cservenka et al., 2015) and that cognitive control function is maximized when FPN and DMN are anti-correlated (Kelly et al., 2008; Hampson et al., 2010; Keller et al., 2015), we hypothesized that girls would: (2) stronger functional connectivity between FPN and DMN and worse performance during cognitive control trials in SRP (but not Control) conditions, compared to boys.

## Materials and Methods

Healthy adolescents were recruited through fliers distributed in the local community. Based on the current aims, only adolescents between the ages of 15 and 18 years who were enrolled in high school were targeted. Youth assent/consent and parent consent (for minors) was obtained for all participants. All procedures were approved by the Oregon Health & Science University Institutional Review Board and were in accordance with the Code of Ethics of the World Medical Association. All subjects gave written informed consent in accordance with the Declaration of Helsinki. Youth and parents were compensated $110 and $60, respectively, for their participation. Data were collected on 49 adolescents (girls = 25). Boys and girls did not differ based on age, pubertal development, IQ, socioeconomic status (SES) or racial distribution (Table 1). Behavioral data are reported for 48 adolescents (see “Behavioral Responding During SRP and Control Trials” section), while functional connectivity results represent data from 40 youth (see “Functional Connectivity” section).

**Table 1:** Participant characteristics.

	**Girls (*n* = 25)**	**Boys (*n* = 24)**	**All (*N* = 49)**	**Statistic**	***p*-value**
Age (Mean ± SD)	16.3 ± 0.9	16.7 ± 0.9	16.5 ± 0.9	*t*_(47)_ = −1.54	0.13
Tanner stage (4/5)	7/18	5/19	12/37	*χ^2^*_(1)_ = 0.34	0.56
IQ (Mean ± SD)^a^	111.3 ± 9.7	113.9 ± 10.1	112.6 ± 9.9	*t*_(47)_ = 0.36	0.36
SES (Mean ± SD)^b^	27.2 ± 14.5	25.6 ± 12.8	26.4 ± 13.6	*t*_(47)_ = 0.40	0.69
White (%)^c^	88	92	90	*χ*^2^_(2)_ = 0.98	0.61

*^a^Wechsler Abbreviated Scale of Intelligence (WASI; 2-subtest version); ^b^Hollingshead Index of Social Position; lower values indicate higher socioeconomic status (SES); means for girls and boys correspond to upper middle class; ^c^Race was coded as 1 = “American Indian/Alaska Native,” 2 = “Asian,” 3 = “Native Hawaiian/Other Pacific Islander,” 4 = “Black or African American,” 5 = “White,” 6 = “Multiple,” and 7 = “Spanish/Hispanic/Latino;” however, only codes 4, 5 and 6 represented the current sample; SD = standard deviation*.

### Exclusionary Criteria

The Diagnostic Interview for Children Predictive Scales (DPS) was administered to the youth and their parent to exclude for the presence of current psychiatric disorders in youth based on Diagnostic Statistical Manual-IV criteria (Lucas et al., 2001). Youth who reported >3 lifetime alcohol binge occasions (≥4 drinks per occasion for females and ≥5 drinks per occasion for males), any alcohol binge occasion in the past 6 months, >20 lifetime uses of marijuana, any marijuana use in the past 6 months, smoking >4 cigarettes per day, or any other drug use were excluded from the study. Additional exclusionary criteria included Tanner pubertal stage ≤3; current home-schooling; DSM-IV Axis I psychotic disorder in either biological parent (e.g., bipolar I or schizophrenia); parent-reported prenatal exposure to alcohol/drugs; serious medical condition(s), including significant head trauma (loss of consciousness ≥2 min); learning disability; inability of parent to provide family history information; current use of psychotropic medications; premature birth (<36 weeks); uncorrected vision problems; magnetic resonance imaging (MRI) contraindications (e.g., braces or claustrophobia); left-handedness (Oldfield, 1971); and pregnancy.

### Participant Characterization

To obtain an estimate of intelligence, youth completed the 2-subtest version of the Wechsler Abbreviated Scale of Intelligence (WASI; Marini et al., 2016). Pubertal development was estimated with the modified line drawing version of Tanner’s Sexual Maturation Scale (Taylor et al., 2001). To measure self-reported depression and anxiety symptoms, participants completed the Children’s Depression Inventory (CDI; Kovacs, 1985) and the state anxiety component of the Spielberger State-Trait Anxiety (STAI) for Children (Spielberger et al., 1983), respectively. Parents completed the Hollingshead Index of Social Position (Hollingshead, 1975) to estimate the SES of the family.

To assess prosocial orientation, youth completed the Shame-proneness and Guilt-proneness subscales of the Test of Self-Conscious Affect for Adolescents (TOSCA-A), which inhibit and promote prosocial behavior, respectively (Tangney et al., 1991) and the Prosocial Tendencies Measure (PTM), which provided information about the contexts that elicit prosocial behaviors (Carlo and Randall, 2002). The Social Competence, Close Friendships and Global Self Worth sub-scales of the Self-Perception Profile for Adolescents (SPP-A; Harter et al., 1998) quantified social self-perceptions. The Co-Rumination Questionnaire (CRQ), which assessed the degree to which an individual engages in repetitive and compulsive conversations that have a negative focus with a same-gender peer (Rose, 2002) was used to quantify potentially maladaptive patterns of peer engagement.

### Self-Referential Processing (SRP)-Flanker Task

**Figure 1:**
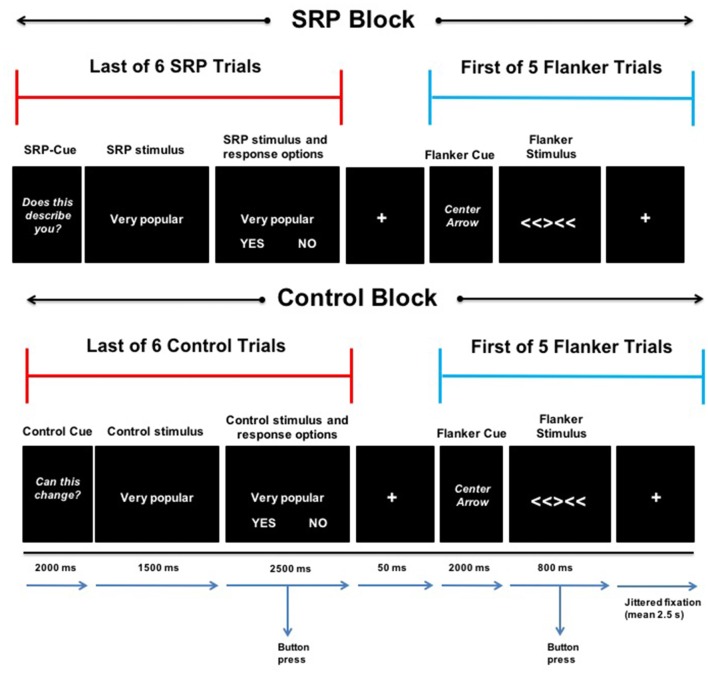
Self-referential processing (SRP)-Flanker Task. The last of six consecutive SRP (top) and Control (bottom) trials (separated by jittered fixation; mean 2.5 s) is presented next to the first of five consecutive Flanker trials (separated by jittered fixation; mean 2.5 s) during an SRP (top) and Control (bottom) block. SRP and Control blocks included a 2-s cue, followed by 4-s stimulus with the options “YES” and “NO” during the last 2.5 s. During SRP blocks, participants indicated with a button press whether the stimulus phrase described them (yes or no; top). In the case of Control trials, participants indicated whether the stimulus phrase can change in other people (yes or no; bottom). A 50-ms fixation separated the last SRP and Control trials from the first Flanker trial of SRP and Control blocks, respectively. Flanker trials began with a 2-s cue, followed by a 0.8-s stimulus presentation of an incongruent or congruent (not shown) condition, during which participants indicated the direction the center arrow was pointing (left or right).

The SRP-Flanker task used a mixed block/event-related design and combined a version of a previously published self-referential task (Jankowski et al., 2014) and a version of the Eriksen flanker task (Eriksen and Eriksen, 1974) that utilizes arrows (Casey et al., 2000; Bunge et al., 2002; Fan et al., 2002; Kelly et al., 2008). Two types of blocks were presented that either consisted of: (1) SRP trials immediately followed by Flanker trials; or (2) Control trials immediately followed by Flanker trials. Blocks contained six SRP or Control trials (4 s each), immediately followed by presentation of five Flanker trials (0.8 s each). Blocks (four SRP and four Control) were presented for approximately 38 s and separated by 12 s of fixation. Presentation of SRP and Control blocks alternated and was counterbalanced across participants for which type of block was first presented. The onset of SRP, Control and Flanker trials was preceded by a 2 s cue: “DOES THIS DESCRIBE YOU?”, “CAN THIS CHANGE?”, and “CENTER ARROW,” respectively (Figure 1). The task was presented with E-Prime 2.0 software (Psychology Software Tools, Pittsburgh, PA, USA) and responses were recorded for all trials. Prior to scanning, youth were provided with a brief demonstration of the task and allowed to practice.

SRP and Control blocks contained the same stimuli, but participants were instructed to respond differently based on the condition. Participants viewed positively and negatively valenced trait phrases representing academic, physical and social domains (Jankowski et al., 2014). During SRP trials, participants were instructed to respond based on whether a given phrase described them (Jankowski et al., 2014). In contrast, in the Control trials, participants were instructed to make evaluations based on the malleability of the same traits with respect to people in general and not themselves (Jankowski et al., 2014). Stimuli were presented in a randomized order, regardless of valence and domain, and each stimulus was presented once per condition. In the scanner, participants were presented with a stimulus in the center of the screen for 1.5 s then the words “YES” and “NO” were presented below the stimulus phrase for 2.5 s during which participants made a button-box response. “YES” was always presented in the bottom left corner of the screen and “NO” was always presented in the bottom right corner of the screen. Presentation of SRP and Control trials was jittered (mean = 2.5 s); participants viewed a fixation cross at the center of the screen between trials.

During Flanker trials, participants viewed a row of five arrows pointing right or left. The four flanking arrows all pointed the same direction (left or right); however, the center arrow pointed in a congruent direction (40%) or incongruent direction (60%). Flanker trials were jittered (mean = 2.5 s) with fixation between trials (Figure 1). Congruent and incongruent trials were presented randomly within blocks. Participants were instructed to make a response to indicate whether the center arrow was pointing left or right, regardless of where the flanking arrows were pointing. They were asked to respond as quickly and as accurately as possible during the 0.8 s display of the arrows. Flanker trials were presented rapidly to increase attentional demands and decrease the likelihood of self-referential thoughts during these trials. As a reminder, the goal of this study was not to examine task-related activation *per se*, but rather to examine functional connectivity (following task regression) of DMN and FPN regions of interest during Flanker trials that followed SRP vs. control conditions.

### Image Data Acquisition

A 3T Siemens Magnetom Tim Trio (Siemens Medical Solutions, Erlangen, Germany) and 12-channel head coil were used to collect imaging data. First, a whole-brain, high-resolution T1-weighted MPRAGE sequence was collected in the sagittal plane (TR = 2300 ms, TE = 3.58 ms, TI = 900 ms, matrix = 240 × 126, FOV = 240 mm, flip angle = 10°, 160 contiguous slices, resolution 1 × 1 × 1.1 mm, 176 repetitions, 9:14 min) for co-registration to functional data. Functional data were collected in one run using high-angular resolution T2*-weighted echo-planar blood-oxygen-level dependent (BOLD) sequences in the axial plane oblique to the anterior-posterior commissure with the following parameters: TR = 2000 ms, TE = 30 ms, matrix = 240 × 176, FOV = 256 mm, flip angle = 90°, 33 contiguous slices, resolution = 3.8 mm^3^, 282 repetitions, 9:30 min.

### Single-Subject Analysis

#### Image Preprocessing

Given that the goal of this study was not to examine task-related activity during either the SRP or flanker task trials, but rather to examine task-regressed functional connectivity of DMN and FPN regions of interest following SRP and control conditions, the data underwent a standard processing pipeline that is commonly used in task activation processing, followed by regression of the task signal and additional processing of the residual data commonly used in resting state functional connectivity processing.

**Figure 2:**
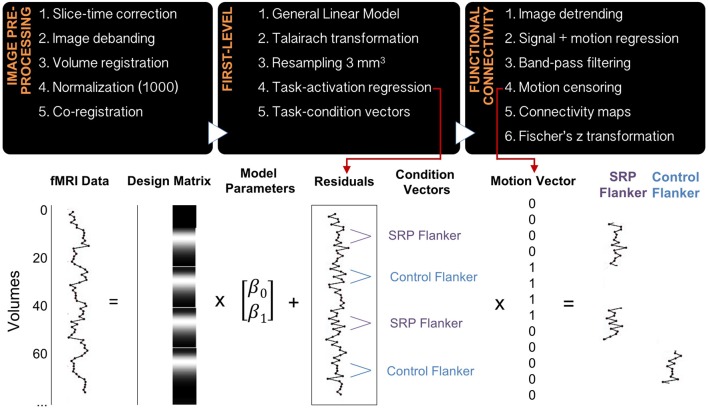
Data preprocessing and analysis pipeline. The diagram includes the steps for image preprocessing, first-level analysis and function connectivity analysis (top), as well as a schematic of a general linear model being applied to the raw imaging data and residual time courses undergoing additional preprocessing, motion censoring and grouping into epochs that represent SRP and Control Flanker blocks (bottom). Averages of similar epochs were used to create functional connectivity maps.

Data were processed using in-house software that implemented 4dfp tools developed at Washington University and previously published (Fair et al., 2012; Alarcón et al., 2015). Functional data processing steps included slice-time correction, image debanding, volume registration, including a 6-parameter rigid body motion realignment, normalization to a mode value of 1000 and co-registration to the anatomical file. The first four frames of functional data were excluded to allow BOLD signal to reach steady state, and the remaining data were modeled in native space. A general linear model (GLM) using a mixed block/event-related design was implemented to analyze the functional task data. SRP and Control blocks were modeled with boxcar functions, while SRP, Control and Flanker events were modeled without an assumed response shape. Flanker congruent and incongruent trials were modeled separately, as were error trials, task instruction trials and start cues for SRP and Control blocks. Next, data were transformed to Talairach standard space and resampled to a 3 mm^3^ resolution. Residual files were created from the GLM by removing all modeled effects, including linear trends and the baseline. Residual files underwent additional processing, including image detrending, multiple regression of whole-brain signal, white matter signal, cerebrospinal fluid signal and their derivatives, as well as 24 motion-related regressors (Friston et al., 1996) and band-pass filtering (0.009–0.08 Hz; Figure 2). Regressing the 24 motion parameters from the residual files, despite having previously regressed the six rigid-body motion parameters in the GLM analysis, was performed due to previous findings that functional connectivity analyses are particularly sensitive to in-scanner micro-movements, particularly in developmental samples (Satterthwaite et al., 2012).

Two vectors, each representing frames of data during SRP Flanker and Control Flanker trials, were created for each subject. A different, motion censoring vector representing usable frames of data, based on a strict motion criterion of frame-to-frame displacement (FD), was also created per subject. The FD method indexes head movement relative to adjacent volumes (Power et al., 2012). Frames were excluded from analysis if they exceeded a threshold of 0.5 mm, and uncensored segments of data with fewer than five contiguous frames were subsequently censored as well. This threshold was determined based on research showing that motion scrubbing thresholds for task fMRI data are most effective within the range 0.5–1.1 mm (Siegel et al., 2014); to err on the conservative side, a 0.5 mm threshold was selected. The SRP Flanker vector and the Control Flanker vector were multiplied with the motion-censoring vector. The products of these vectors included: (1) frames of data with minimal motion from SRP Flanker trials; and (2) frames of data with minimal motion from Control Flanker trials. An FD remaining mean variable was calculated for every individual, representing the degree of micro-movement (in the range of millimeters) of this remaining motion-censored data (Figure 2).

#### Creating Functional Connectivity Maps

Bilateral dorsolateral prefrontal cortex (DLPFC) is a key node of the FPN (Cole and Schneider, 2007), while medial prefrontal cortex represents a hub of the DMN. Representative coordinates were selected from a functionally-defined set of ROIs based on several meta-analyses of task fMRI data and functional connectivity mapping (Cohen et al., 2008; Power et al., 2010, 2012), where ROIs are modeled as 10 mm diameter spheres centered upon ROI coordinates (Power et al., 2011). FPN and DMN coordinates that most closely matched those reported in FPN (Cole and Schneider, 2007) and DMN in the context of SRP (Northoff et al., 2006) were selected and their functional associations were confirmed on neurosynth.org. Refer to Table 2 for final selection of seed regions. Time courses from every seed region per condition (SRP Flanker and Control Flanker) were extracted and correlated with every voxel in the brain, generating correlation coefficients that underwent a Fisher’s *z* transformation to improve data normality (Figure 2).

**Table 2:** Functional connectivity analysis seed regions.

	Peak Brodmann area	Peak Talairach coordinates (*X, Y, Z*)
Dorsolateral prefrontal cortex
Left	46	−41, 33, 24
Right	8	37, 13, 42
Medial prefrontal cortex
Left	9	−8, 42, 27
Right	10	8, 48, 9

### Group-Level Analysis

#### Participant Characterization

Statistical analyses were carried out with IBM SPSS Statistics 24 (Armonk, NY: IBM Corp). Gender differences in demographic variables were examined with independent samples *t*-tests, Mann-Whitney U analysis or Chi-square analysis, as appropriate. Gender differences in FD remaining mean were assessed with independent samples *t*-tests. Reaction time (RT), errors of omission and accuracy on Flanker trials were analyzed with repeated measures analysis of variance (ANOVA; congruent vs. incongruent). Gender difference in responding during SRP and Control trials was also examined with repeated measures ANOVA (Positive Academic vs. Negative Academic vs. Positive Social vs. Negative Social vs. Positive Physical vs. Negative Physical). Greenhouse-Geisser correction was applied as appropriate, and *post hoc* analyses were controlled for multiple comparisons using Šidák correction. Spearman correlations between self-reported measures, SRP and Control Flanker performance and significant functional connectivity findings were conducted. Multiple comparisons correction for correlation tests was accomplished with the Benjamini-Hochberg procedure (Benjamini and Hochberg, 1995) and reported as “corrected,” unless otherwise noted.

#### Functional Connectivity Analyses

Whole-brain, task-regressed functional connectivity analyses were conducted with Analysis of Functional NeuroImages (AFNI; version 16.2.07; Cox, 1996) using 2 (SRP vs. Control) × 2 (girls vs. boys) ANOVAs for each FPN and DMN seed region. Congruent and Incongruent trials of the Flanker task were collapsed within SRP and Control conditions to preserve detection power. Importantly, we found that patterns of task activation of Flanker congruent and incongruent trials did not differ significantly (data not shown). Furthermore, because we used a 3:2 ratio for incongruent and congruent trials—compared to larger ratios (e.g., 4:1 or 3:1)—the differences in activation are minimized and reflective of more proactive attentional control, rather than reactive attentional control, which additionally engages frontal and inferior temporoparietal cortices (Marini et al., 2016). A voxel-wise threshold of *p* < 0.01 and a cluster-wise threshold of *p* < 0.05 was implemented using AFNI’s 3dClustSim (-NN 1, 2-sided) after estimating data noise smoothness values with 3dFWHMx, leading to a minimum cluster size of 44 voxels. Functional connectivity values from significant clusters were extracted to test *post hoc* effects plotted in figures; multiple comparisons correction was completed with Šidák correction.

#### Confirmation of Task Effects Modeling

To confirm that the effects of the task were successfully modeled and removed for task-regressed functional connectivity analyses, residualized files were submitted to a whole-brain voxel-wise two-way ANOVA (within: SRP vs. Control, between: female vs. male) in AFNI. A minimum cluster size was determined using the same procedure implemented in functional connectivity analyses, with a voxel-wise threshold of *p* < 0.05 and a cluster-wise threshold of *α* < 0.05 (minimum cluster size ≥10 voxels). Residualized files contained less noise than task activation files; therefore, resulting in smaller smoothness estimates and a smaller minimum cluster size to reach statistical significance.

## Results

### Participant Characterization

Mann-Whitney U analyses indicated that girls reported higher mean co-rumination scores than boys (Girls = 3.0 ± 0.8; Boys = 2.1 ± 0.8; *U* = 117.0, *p* = 0.007, corrected); however, significant gender differences in other social measures did not emerge (*p* > 0.05, corrected). Independent samples *t*-tests indicated that depression sub-scale and total T-Scores and state anxiety scores were not different by sex (*p* > 0.05, corrected).

### Behavioral Responding During SRP and Control Trials

Behavioral data from SRP and Control trials were missing from one (female) individual due to a technical problem. For SRP trials, there was a significant main effect of domain (*F*_(3.77,169.67)_ = 22.93, *p* < 0.001, Greenhouse-Geisser correction), but no significant main effect of gender (*F*_(1,45)_ = 0.87, *p* = 0.36) or interaction of domain and gender (*F*_(3.77,169.67)_ = 1.43, *p* = 0.23, Greenhouse-Geisser correction). *Post hoc* analysis indicated that youth believed Positive Academic traits described them more than Negative Academic, Negative Social, Positive Physical and Negative Physical traits (all *p* ≤ 0.0004, Šidák). Further, youth agreed that Positive Social traits described them more than Negative Academic, Negative Social, Positive Physical and Negative Physical traits (all *p* ≤ 0.02, Šidák). Lastly, Positive Physical traits were endorsed more than Negative Physical traits (*p* = 0.007, Šidák). Thus, overall youth agreed that positive traits described them more than negative traits.

Similarly, a repeated measures ANOVA indicated a main effect of domain in the Control condition (*F*_(5,225)_ = 14.32, *p* < 0.001). The main effect of domain was driven by an overall difference in Positive Academic traits, such that youth believed Positive Academic traits were the least malleable, compared to all other traits (all *p* < 0.0001, Šidák). The main effect of gender (*F*_(1,45)_ = 0.14, *p* = 0.71) and the interaction of domain and gender (*F*_(5,225)_ = 1.16, *p* = 0.33) were not significant.

### Behavioral Responding During Flanker Trials

**Figure 3:**
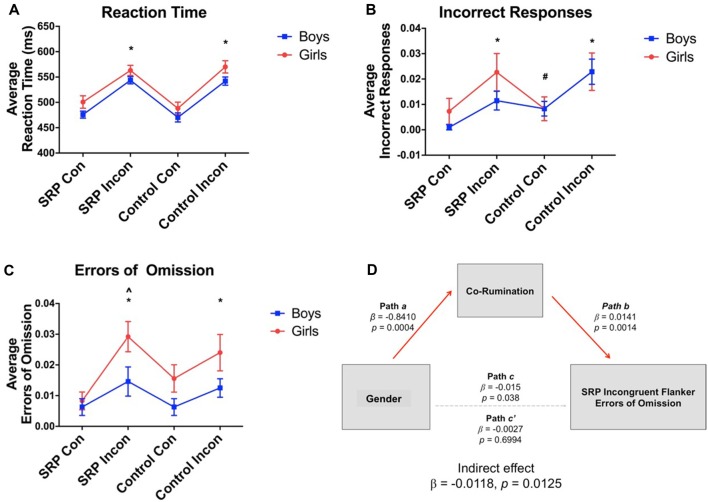
Flanker task performance (mean ± standard error of the mean; *N* = 49). Reaction time (RT) was slower **(A)** and more incorrect responses **(B)** and errors of omission **(C)** were made during incongruent vs. congruent trials, regardless of condition, as indicated by significant main effects of condition. Main effects of gender on RT and errors of omission were also observed, such that girls were slower to respond and made more errors than boys. Co-rumination fully mediated the effect of gender on SRP Incongruent errors of omission **(D)**. *Compared to SRP Congruent and Control Congruent trials, *p* < 0.05, Šidák. ^#^Compared to SRP Congruent trials, *p* < 0.05, Šidák. ^∧^Girls compared to boys, *p* < 0.05, Šidák. SRP, self-referential processing; Con, Congruent; Incon, Incongruent.

Behavioral data were missing from one (female) individual due to a technical problem. Repeated measures ANOVAs showed significant main effects of trial type for RT (*F*_(3,138)_ = 56.32, *p* < 0.001), accuracy (*F*_(2.06,94.73)_ = 8.59, *p* < 0.001, Greenhouse-Geisser correction) and errors of omission (*F*_(3,138)_ = 6.46, *p* < 0.001). Overall, participants were slower to respond during Incongruent vs. Congruent trials, regardless of SRP or Control condition (Figure 3). A significant main effect of gender emerged for RT (*F*_(1,46)_ = 4.69, *p* = 0.04) and errors of omission (*F*_(1,46)_ = 6.84, *p* = 0.01), but not accuracy (*F*_(1,46)_ = 0.74, *p* = 0.40). In support of our hypothesis, girls were slower to respond regardless of trial type and committed more errors of omission during SRP Incongruent trials than boys, but not the remaining trial types (*p* < 0.05, Šidák; Figure 3). Finally, interactions between gender and trial type for RT (*F*_(3,138)_ = 0.19, *p* = 0.91), accuracy (*F*_(2.06,94.73)_ = 0.89, *p* = 0.42, Greenhouse-Geisser correction) and errors of omission (*F*_(3,138)_ = 1.03, *p* = 0.38) were not significant.

Spearman correlations indicated that errors of omission during SRP Incongruent trials were positively correlated with co-rumination scores (*ρ* = 0.56, *p* = 0.006, corrected). When examined by sex, co-rumination was positively correlated with errors of omission in boys (*ρ* = 0.57, *p* = 0.004), but not girls (*ρ* = 0.37, *p* = 0.07); however, the correlation coefficients were not significantly different by sex (*z* = 0.86, *p* = 0.19, one-tailed). Given gender differences in co-rumination mean scores, a mediation analysis was pursued to examine whether co-rumination mediated gender differences in SRP Incongruent omissions. Mediation was performed with the PROCESS macro (Hayes, 2013) in SPSS, with gender as the independent measure, SRP Incongruent omission errors as the dependent measure and a co-rumination as the mediator. Bias corrected bootstrapped 95% confidence intervals were determined with 5000 bootstrapped samples. The direct effect of gender on omissions became non-significant once co-rumination was included in the model, while the indirect pathway was statistically significant, indicating that co-rumination fully mediated the effect of gender on SRP Incongruent omissions (Figure 3).

### Functional Connectivity

Based on the strict motion censoring criteria, 9 of 49 participants had zero frames of data and could not be included in functional connectivity analyses. These nine participants did not differ from the rest of the sample on any demographic, clinical or behavioral variables (all *p* > 0.05). Independent samples *t*-tests indicated that the number of frames of data used in the imaging analysis was not significantly different by gender for either the SRP (Girls = 25.00 ± 7.84 [range: 7–33]; Boys = 23.11 ± 9.93 [range: 7–34]; *t*_(38)_ = 0.82, *p* = 0.42) or Control (Girls = 25.25 ± 8.62 [range: 7–33]; Boys = 22.79 ± 10.19 [range: 7–34]; *t*_(38)_ = 0.80, *p* = 0.43) conditions out of 40 possible frames for each condition. On average, the same number of frames was analyzed in SRP vs. Control conditions per subject (SRP = 24.12 ± 8.77; Control = 23.95 ± 9.17; *t*_(39)_ = 0.04, *p* = 0.97). Boys and girls did not differ on mean FD of the remaining frames for SRP (Girls = 0.10 ± 0.04; Boys = 0.11 ± 0.03; *t*_(38)_ = 0.52, *p* = 0.61) or Control (Girls = 0.10 ± 0.03; Boys = 0.12 ± 0.04; *t*_(38)_ = 1.66, *p* = 0.11) conditions.

#### Frontoparietal Network (FPN)

**Figure 4:**
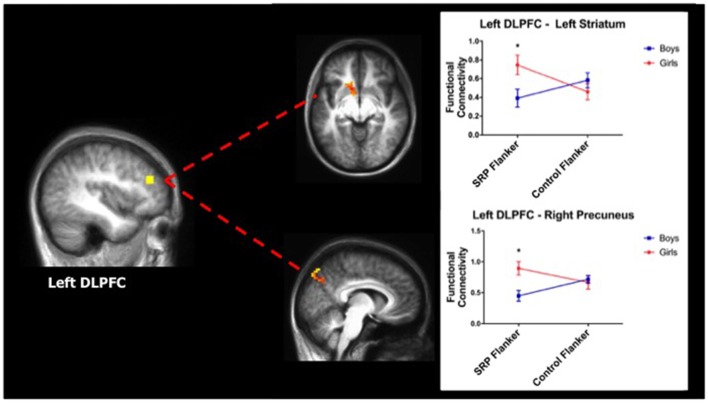
Girls showed stronger functional connectivity during SRP Flanker trials (mean ± standard error of the mean; *n* = 40). Left dorsolateral prefrontal cortex (DLPFC) was more strongly functionally connected to left striatum (top) and right precuneus (bottom), a region of the default mode network (DMN), during SRP Flanker vs. Control Flanker conditions in girls, compared to boys. Functional connectivity during Control Flanker trials was not different by gender. *p < 0.05, Šidák.

A two-way ANOVA of DLPFC functional connectivity yielded significant effects with the left DLPFC seed region only. Specifically, significant interactions between gender and condition emerged in left ventral/dorsal striatum and right precuneus (region of the DMN), such that girls showed stronger functional connectivity between these regions and the left DLPFC during SRP, compared to boys. *Post hoc* analysis revealed that girls had stronger functional connectivity between left DLPFC and both striatum and precuneus during SRP Flanker trials (*p* < 0.05, Šidák), but not Control Flanker trials (*p* > 0.05, Šidák), compared to boys (Table 3; Figure 4).

**Table 3:** Functional connectivity results (*n* = 40).

Seed region	Functionally coupled region	BA	Voxel number	Peak Talairach coordinates (*X, Y, Z*)	Cohen’s *d*^a^
**Main effect of gender**
L Medial prefrontal cortex	L Posterior cerebellum		65	32, −84, −27	0.48
**Condition-by-gender interaction**
L Dorsolateral prefrontal cortex	L Ventral/Dorsal striatum		49	−10, 9, −6	0.39
	R Precuneus	7	46	8, −75, 39	0.40
R Medial prefrontal cortex	R Dorsolateral prefrontal cortex	9/10	70	38, 36, 18	0.38

*^a^Cohen’s d effect sizes range from small (*d* = 0.2), medium (*d* = 0.5) and large (*d* = 0.8); BA, Brodmann Area; L, left; R, right*.

#### Default Mode Network (DMN)

**Figure 5:**
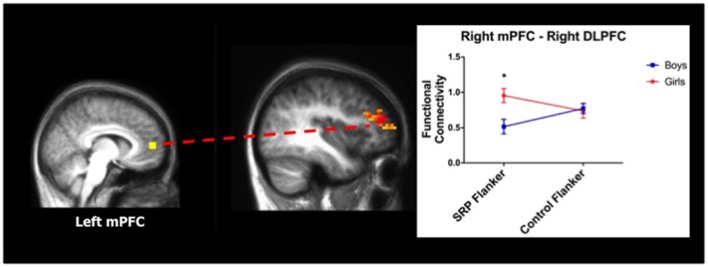
Girls showed stronger functional connectivity during SRP Flanker trials (mean ± standard error of the mean; *n* = 40). Right medial prefrontal cortex (mPFC) was more strongly functionally connected to right DLPFC, a region of the frontoparietal network (FPN), during SRP Flanker trials vs. Control Flanker trials in girls, compared to boys. Functional connectivity during Control Flanker trials was not different by gender. *p < 0.05, Šidák.

A two-way ANOVA of left mPFC functional connectivity indicated a significant main effect of gender with right posterior cerebellum, with girls showing stronger functional connectivity than boys. Moreover, in the right mPFC seed analysis, a significant interaction between gender and condition was found with right DLPFC functional connectivity, as indicated by two-way ANOVA. Girls had stronger functional connectivity between mPFC and right DLPFC during the SRP Flanker condition (*p* < 0.05, Šidák), but not Control Flanker condition (*p* > 0.05, Šidák), compared to boys (Figure 5; Table 3).

#### Correlations

Spearman correlations indicated that mean co-rumination scores were not significantly correlated to functional connectivity values across the sample or by gender (*p* > 0.05, corrected). Likewise, SRP and Control Flanker performance was not significantly correlated with functional connectivity values across the sample or by gender (*p* > 0.05, corrected).

### Confirmation of Task Effects Modeling

Following analysis with two-way ANOVA, there were no significant main effects of sex or condition (SRP vs. Control) or significant interactions between sex and task condition using residualized task files, indicating that the task was effectively modeled and the task-regressed residual files used in functional connectivity analyses represent underlying signal fluctuations that have been influenced by task conditions.

## Discussion

The primary aims of this study were to examine gender differences in performance and functional connectivity between DMN and FPN during conditions that placed internal (i.e., SRP) and external (i.e., cognitive control) attentional demands in conflict. In partial support of our hypotheses, compared to boys, girls demonstrated more interference on cognitive control performance in the SRP context, as reflected by more errors of omission during SRP Incongruent trials. As hypothesized, girls displayed stronger functional connectivity of FPN and DMN during SRP Flanker trials relative to boys. Finally, co-rumination, which was the only self-reported measure that differentiated boys and girls, mediated cognitive control performance during SRP Incongruent conditions. The outcomes of this study suggest that placing SRP and cognitive control processes in conflict is reflected by stronger functional connectivity of DMN and FPN, as well as poorer cognitive control performance in girls, compared to boys; however, co-rumination (or possibly other related, but unmeasured aspects of prosocial behavior) may explain a larger portion of cognitive control performance than functional connectivity.

### Cognitive Control Performance

Performance on the Flanker trials reflected that of previous studies, where participants were slower to respond during incongruent relative to congruent trials (Casey et al., 2000; Bunge et al., 2002; Fan et al., 2002; Wager et al., 2005; Kelly et al., 2008; Mennes et al., 2011). This pattern was observed within both SRP and Control conditions. SRP and Control Incongruent trials were the most difficult, as reflected by slower RT, than all congruent trials. However, there were no differences in difficulty between the two types of incongruent trials, nor the two types of congruent trials. Similar patterns were observed with accuracy, which also reflects task difficulty, and omissions, which more closely represent lapses in attention (Matthews et al., 2000). These findings may indicate that, for the most part the SRP manipulation did not increase the difficulty of these trials. However, girls responded more slowly than boys across all conditions, perhaps because they perceived the entire task as more difficult, or perhaps because they were more deliberate in their responding. The design of this study does not provide a means to differentiate between response strategies; thus, future studies may consider parametric modulation of task difficulty or retrospective self-report as ways to address this question. Importantly, *post hoc* analysis of significant main effects of gender revealed a condition-specific gender difference in performance, as indexed by errors of omission, such that girls made more errors than boys during SRP Incongruent Flanker trials. Therefore, there is some evidence that the higher rates of omission errors in girls are due to SRP interfering with performance of the most difficult type of Flanker trial (i.e., incongruent), perhaps by increasing executive load (Luciana, 2016). It is also possible that the effect of SRP on cognitive control performance was more pervasive for girls and persisted across all conditions, contributing to their slower RTs and higher rates of omission errors overall. The design of this study permitted a temporal dissociation of SRP and Control conditions of approximately 1 min; however, that may not have been sufficient time for the influence of SRP to dissipate, particularly for girls.

### Functional Connectivity

In accordance with the hypotheses of this study, healthy adolescent girls demonstrated stronger functional connectivity between DMN and FPN during cognitive control trials in SRP conditions, as compared to boys. These findings indicate that the experimental manipulation was effective at differentiating boys’ and girls’ neural responses; however, as stated in “Cognitive Control Performance” section, Flanker task performance outcomes provide mixed support on this front. Notably, functional connectivity was not correlated with Flanker performance or co-rumination, indicating that functional connectivity may not play as large of a role in moderating gender differences in Flanker performance during trials that are presumed to be most difficult (i.e., SRP Incongruent).

Stronger DMN—FPN functional connectivity during SRP Flanker trials was reflected by DLPFC—precuneus and mPFC—DLPFC functional connectivity. As a node of the DMN, the precuneus is involved in episodic memory and self-reflection (Cavanna and Trimble, 2006). Moreover, functional connectivity between precuneus and DLPFC, as well as mPFC and DLPFC, has been shown to be positively linked with self-related sharing (Meshi et al., 2016), which may indicate that girls’ self information brought online during SRP trials remained in working memory and competed for resources with cognitive demands of the Flanker trials, leading to poorer performance. A few brain regions outside of FPN and DMN were also identified in functional connectivity analyses. Compared to boys, girls showed stronger functional connectivity between left mPFC and the right posterior cerebellum. This particular region of the cerebellum has been repeatedly implicated in theory of mind and higher-order social cognition (Carrington and Bailey, 2009; Spunt and Adolphs, 2014; Van Overwalle et al., 2014), suggesting that its connectivity with mPFC, a key region for SRP, may support integration of social information with self-identity—a mechanism that may be more streamlined in girls compared to boys. In addition, girls displayed stronger frontostriatal connectivity (left DLPFC—dorsal/ventral striatum) during SRP, but not Control Flanker trials compared to boys, perhaps as a means to compensate for the ineffective reduction in functional connectivity of DMN and FPN nodes. Indeed, the DLPFC—striatal circuit, in particular, is crucial for intact executive functioning (Alexander et al., 1990; Hampshire et al., 2012).

### SRP Behavior and Co-rumination

Adolescents in this study were more likely to endorse positive than negative qualities about themselves, in agreement with previous studies in early adulthood (Watson et al., 2007; Zhang et al., 2013; Chen et al., 2014; Yang et al., 2014). Gender differences in responding were not observed, indicating that boys and girls did not differ in their self-perceptions about emotionally-salient traits across domains of life that are relevant to adolescents (i.e., academic, social and physical). This conclusion is supported by the lack of sex differences in self-perception as measured by the SPP-A Social Competence and Global Self-Worth sub-scales. Despite a lack of gender differences in self-perception, girls reported more co-rumination than boys, which mediated the effect of gender on SRP Incongruent Flanker omissions. During adolescence, co-rumination predicts increases in depressive symptoms by way of increasing rumination (Stone and Gibb, 2015), as well as mediates the effect of gender on depressive symptoms (Rose, 2002). Thus, not only does co-rumination reflect a pattern of peer engagement that is potentially maladaptive, but it also indicates the presence of negative cognitive style that increases vulnerability for depression. Given that adolescent girls experience more social-evaluative concern (Rudolph and Conley, 2005), being asked to reflect on their self-identity, which reflexively incorporates the perceptions of peers, likely leads to a more pervasive sustainment of internally-focused attention in girls compared to boys. In the current study, youth who tended to engage in more co-rumination (i.e., girls) may have been more likely to perseverate on SRP processes, which may function to increase cognitive load during Flanker trials and decrease performance. Notably, co-rumination was not correlated with DMN—FPN functional connectivity, suggesting that any potential effects on cognitive load would be unique from connectivity of these specific networks.

### Strengths, Limitations and Alternative Interpretations

Strengths of this study include a well-characterized sample and rigorous data processing that included motion scrubbing. Girls and boys were well matched on various demographic and developmental variables, which served to eliminate potential confounds that could explain the current findings. However, one limitation of this work was the relatively small sample size, which was especially relevant given that functional connectivity analyses only includes frames of data from Flanker trials that did not exceed the motion threshold (SRP average = 24.12; Control average = 23.95) This analytical approach greatly reduced the amount of data used for analysis, albeit, to a similar degree in males and females. However, gender differences in functional connectivity were observed with medium effect sizes, indicating that there was sufficient power to detect meaningful effects.

It is notable that results were obtained for left DLPFC for the FPN analyses and that connectivity with mPFC was found with right DLPFC for the DMN analyses. The lateralization of DLPFC findings may be due to the fact that DLPFC seed regions were not centered around the exact ipsilateral coordinates. DLPFC seed regions were part of the FPN network (Power et al., 2011) and we confirmed that they were associated with cognitive control processing through neurosynth.org. There is support for this interpretation, given that degree of functional connectivity between DLPFC and mPFC seed regions and their corresponding networks, FPN and DMN, respectively, appears to vary based on hemisphere (neurosynth.org). In the case of DLPFC, the left DLPFC seed region qualitatively appears to be better integrated with the FPN than the right DLPFC seed region. In the case of mPFC, the right seed region qualitatively appears to be better integrated with the DMN than the left mPFC seed region. Thus, we believe, lateralized effects are a result of seed region definition that may be overcome with larger sample sizes.

As a limitation, the design of the current study did not permit analysis of SRP functional connectivity based on the domains and/or valence of the SRP statements. In every SRP and Control block, statements were presented randomly and consecutively in order to sustain an SRP effect that would carry over to the subsequent set of Flanker trials. Thus, the effects of domain and valence across SRP trials represent averages and their unique effects cannot be measured. It is possible that either valence or domain of the SRP phrases may have differentially influenced SRP functional connectivity and its sustained effect on Flanker trials. Given that the majority of adolescents in the present study reported identifying with positive vs. negative phrases, it is possible we did not capture the effects of negative SRP. Future studies may consider personalizing statements to reflect negative self-relevant thoughts of each individual (Carew et al., 2013) or separating positive, negative and/or non-affective SRP conditions to examine their unique effects.

In spite of numerous gender differences in functional connectivity, the number of behavioral gender differences in the current study was very sparse. Indeed, a group difference in omissions during SRP Incongruent Flanker trials was the only finding where boys and girls differed in their responding in a context specific to SRP. Notably, this finding, which was pursued due to* a priori* hypotheses, was detected *post hoc* despite a lack of a significant interaction between gender and task condition. Therefore, this particular finding must be interpreted with caution. It is possible that gender differences in Flanker performance are not as robust as gender differences in functional connectivity. Indeed, this may be the case given that the current sample was generally healthy, and gender differences in cognitive control performance are not commonly found in healthy youth despite gender differences in brain activation (Weiss et al., 2003; Schweinsburg et al., 2005; Christakou et al., 2009; Li et al., 2010; Rubia et al., 2010, 2013; Alarcón et al., 2014; Hjelmervik et al., 2014; White et al., 2014; Cservenka et al., 2015).

Functional connectivity, specifically during resting states, is generally considered a relatively stable measure (Braun et al., 2012; Chou et al., 2012; Guo et al., 2012; Song et al., 2012; Franco et al., 2013; Rzucidlo et al., 2013; Hjelmervik et al., 2014; Zuo and Xing, 2014; Chen et al., 2015; Du et al., 2015; Shah et al., 2016) across participants and mental states (Calhoun et al., 2008; Smith et al., 2009; Cole et al., 2014; Krienen et al., 2014), which has made it possible to examine its relationship to behavioral correlates (Fulwiler et al., 2012; Baur et al., 2013; Takeuchi et al., 2013; Modi et al., 2015; Pan et al., 2016). However, recent work demonstrates that there is an appreciable difference in network functional connectivity between task and resting states, such that task-dependent functional connectivity effects explain as much or more of the variance in inter-individual connectivity than resting state effects (Geerligs et al., 2015). Moreover, differences in functional connectivity between individuals are not static, but greatly depend on the mental state during which these measurements are obtained. The results of the present study support this assertion by demonstrating that gender differences in functional connectivity occurred across task conditions. This suggests that consideration must be given to the participant’s mental state when determining under what context the functional connectivity architecture of an individual provides the most meaningful association to the behavioral correlate being studied.

There are a variety of analytic approaches used to measure task-related functional connectivity, including removal of linear task effects with regression (Fair et al., 2007), removal of task-induced variance with independent component analysis (Arfanakis et al., 2000) and psychophysiological interaction (O’Reilly et al., 2012), with the latter options being most effective for block designs. Linear regression of task effects is not restricted by the experimental design as long as task effects are effectively modeled and removed. Indeed, previous work has shown that this approach may not remove all task-related signal (Fair et al., 2007); however, this very signal may represent altered functional connectivity due to task engagement (Lowe et al., 2000; Hampson et al., 2004; Fransson, 2006; Fair et al., 2007; Zhang and Li, 2010, 2012). Even so, the possibility that nonlinear task effects remain and alter correlation coefficients cannot be ruled out.

## Conclusion

Healthy male and female adolescents demonstrated a differential impact of SRP on cognitive control task performance that was paralleled by DMN-FPN functional connectivity. Specifically, induction of an SRP state interfered with the expected pattern of anti-correlation between DMN and FPN, particularly in girls. Importantly, gender differences in this pattern of connectivity were absent in Control conditions, indicating that these effects were due to the SRP induction. Although behavioral effects were small, results indicated that girls’ performance (i.e., errors of omission) suffered more than boys’ during cognitive control trials within an SRP context. Importantly, co-rumination, which was endorsed to a larger degree by girls than boys, mediated the effect of gender on SRP Incongruent Flanker omissions. Thus, placing internal (i.e., SRP) and external (i.e., cognitive control) attentional demands in conflict was reflected by weaker anti-correlation of DMN and FPN. Engaging in co-rumination may be one mechanism through which cognitive control performance declines in situations where adolescents must regulate internal vs. external attentional demands. Future studies must determine the relevance of this mechanism for predicting depression using similar experimental paradigms longitudinally and with adolescents who are at risk for MDD.

## Data Availability

The raw data supporting the conclusions of this manuscript will be made available by the authors, without undue reservation, to any qualified researcher.

## Author Contributions

GA conceived the study and was involved in data collection, analysis and interpretation, as well as drafting the article. JP, DF and BN contributed to the design of the study, data interpretation and critical revision of the article. All authors approved the final version of the manuscript to be published.

## Conflict of Interest Statement

The authors declare that the research was conducted in the absence of any commercial or financial relationships that could be construed as a potential conflict of interest.
